# Supplementary material for: Content-rich biological network constructed by mining PubMed abstracts
Source: BMC Bioinformatics. 2004 Oct 8;5:147. doi: 10.1186/1471-2105-5-147 (PMC528731; doi:10.1186/1471-2105-5-147)
Supplement: Additional File 5 — The original Chilibot query results of the term "long-term potentiation (LTP)" and 22 other terms, limiting the latest references analyzed to the years 1990, 1995, 2000, and 2004. [file 1471-2105-5-147-S5.bz2 › chilibotAdditionalFile5/ltp2004/html/ERK_NMDA.html]

 


 **ERK** and **NMDA** 
  
Found 50 abstracts in PubMed,  **30 abstracts were retrieved and analyzed**.  


---

 Search Google  |
 PDF files only 
|  EDU domain only 

---

**Interactive relationship** (e.g. stimulation, inhibition, etc)

- 17 beta Estradiol E2, by activating Src and  **ERK**  MAP kinases, enhances  **NMDA**  receptor phosphorylation and function.  Ref: 12928058 Neurobiol Aging, 2003
- PTK,  **ERK**  and p38 MAPK contribute to impaired  **NMDA**  induced vasodilation after brain injury.  Ref: 12921870 Eur J Pharmacol, 2003
- Glutamate mediated activation of  **NMDA**  receptors leads to the rapid but transient phosphorylation of  **ERK**  in cultured neurons.  Ref: 12483215 Nat Neurosci, 2003
- These data show that PTK,  **ERK**  and p38 MAPK activation contribute to impaired  **NMDA**  cerebrovasodilation after fluid percussion brain injury.  Ref: 12921870 Eur J Pharmacol, 2003
- **NMDA**  mediated activation of the tyrosine phosphatase STEP regulates the duration of  **ERK**  signaling.  Ref: 12483215 Nat Neurosci, 2003
- We show that  **NMDA**  activates transcription of CREB responsive genes in hippocampal neurons, with  **ERK**  responsible for persistent CREB phosphorylation and CaM kinase IV CaMKIV responsible for phosphorylating the CREB coactivator, CBP.  Ref: 11970865 Neuron, 2002
- Here we show that activation of  **NMDA**  receptors led to activation of STEP, which limited the duration of  **ERK**  activity as well as its translocation to the nucleus and its subsequent downstream nuclear signaling.  Ref: 12483215 Nat Neurosci, 2003
- This effect depended on the activation of  **NMDA**  receptors but was independent of both calcium calmodulin dependent protein kinases CaMK and extracellular regulated kinase  **ERK** .  Ref: 12807435 J Neurochem< MedlineTA>J Neurochem, 2003
- The  **NMDA**  receptor channel blocker, MK 801, also significantly attenuates photic induction of P  **ERK**  and P Elk 1.  Ref: 12684495 J Neurosci, 2003
- DA D2 and glutamate receptors of  **NMDA**  subtypes also participate, albeit to a lesser extent, to THC induced  **ERK**  activation in the striatum, as shown after injection of selective antagonists raclopride and MK801, respectively.  Ref: 11553284 Eur J Neurosci, 2001
- The glutamate induced declustering of Cupidin preceded that of F actin and drebrin, was elicited by  **NMDA**  receptor mediated calcium influx, and was followed by a downstream pathway including MAPK  **ERK**  and protein tyrosine kinase.  Ref: 14511114 J Neurochem, 2003
- Finally, the effects of cannabinoids on  **ERK**  activation were dependent on the activity of glutamate  **NMDA**  receptors in vivo... indicating the existence of several pathways linking CB1 R to the  **ERK**  cascade.  Ref: 12657697 J Neurosci, 2003
- Using a combination of in vivo electrophysiology, biochemistry, pharmacology, and immunohistochemistry, we found the following 1  **ERK**  phosphorylation, including phosphorylation of nuclear  **ERK** , and  **ERK**  phosphotransferase activity are increased markedly, albeit transiently, after the induction of  **NMDA**  receptor dependent LTD at the commissural input to area CA1 pyramidal cells in the hippocampus of anesthetized adult rats.  Ref: 11896145 J Neurosci, 2002
- Exposure of the cerebral cortex to a xanthine oxidase O 2 generating system OX reversed  **NMDA**  10 0 6 M dilation to vasoconstriction but such impairment was partially prevented by the PTK inhibitor, genistein, the MAPK  **ERK**  isoform inhibitor, U0126, and the MAPK p38 isoform inhibitor, SB203580 9 1 and 15 1 vs. 1 1 and 1 1 vs. 5 1 and 9 1% for sham control, OX and OX in the presence of genistein, respectively.  Ref: 12850576 Brain Res, 2003
- The  **NMDA**  Receptor Is Coupled to the  **ERK**  Pathway by a Direct Interaction between NR2B and RasGRF1.  Ref: 14622581 Neuron, 2003
- This could be due to an interaction with the N methyl d aspartate  **NMDA**  receptor complex or with activity of the extracellularly regulated protein kinase  **ERK**  Ras signalling pathway.  Ref: 11856900 Behav Pharmacol, 2001
- Bicuculline enhanced polysynaptic EPSCs are completely eliminated by  **NMDA**  receptor antagonists APV and ketamine, as was the augmented  **ERK**  activation.  Ref: 14664828 Mol Cell Neurosci, 2003
- Moreover, blockade of  **NMDA**  receptor NMDAR did not decrease FAK, MAPK  **ERK** , and CREB phosphorylation although LTP induction was blunted by NMDAR antagonist.  Ref: 12764094 J Neurosci, 2003
- Taken together, we indicate that TGF alpha rescues neurons from  **NMDA**  induced excitotoxicity in mixed cultures through inhibition of t PA activity, involving PAI 1 overexpression by an  **ERK**  dependent pathway in astrocytes.  Ref: 12490542 FASEB J, 2003

**Parallel relationship** (e.g. studied together, co-existance, homology, etc.)

- SynGAP regulates  **ERK**  MAPK signaling, synaptic plasticity, and learning in the complex with postsynaptic density 95 and  **NMDA**  receptor.  Ref: 12427827 J Neurosci, 2002
- We have previously shown that  **NMDA**  receptors can bi directionally regulate activation of extracellular signal regulated kinase, and  **NMDA**  receptor stimulation of PKB in the present study appeared to mirror activation of  **ERK** .  Ref: 12358757 J Neurochem, 2002
- The newly generated neurons showed  **ERK**  phosphorylation by  **NMDA**  administration as seen in surrounding mature neurons.  Ref: 14720221 J Neurochem, 2004
- While results support that  **NMDA**  receptor signaling precludes Akt activation, we did not find evidence to support that it underlies ischemia induced  **ERK**  phosphorylation.  Ref: 12586553 Neurobiol Dis, 2002
- Differential role of PTK,  **ERK**  and p38 MAPK in superoxide impairment of  **NMDA**  cerebrovasodilation.  Ref: 12850576 Brain Res, 2003
- Furthermore, these data indicate a differential role for  **ERK**  and p38 MAPK activation in impairment of  **NMDA**  dilation by O 2 in the brain.  Ref: 12850576 Brain Res, 2003
- Finally, we showed that TGF alpha, by an  **ERK**  dependent mechanism, stimulates the astrocytic expression of PAI 1, a t PA inhibitor, which mediates the neuroprotective activity of TGF alpha against  **NMDA**  mediated excitotoxic neuronal death.  Ref: 12490542 FASEB J, 2003
- However, the p38 MAPK inhibitor, SB203580, prevented  **NMDA**  dilator impairment significantly less than the  **ERK**  MAPK inhibitor, U0126.  Ref: 12850576 Brain Res, 2003
- The specific association of RasGRF1 with the NR2B subunit and study of  **ERK**  activation in neurons with varied content of NR2B suggests that NR2B containing channels are the dominant activators of the  **NMDA**  dependent  **ERK**  pathway.  Ref: 14622581 Neuron, 2003
- Inhibitors of both phosphatidylinositol 3 kinase PI3 kinase and MAPK  **ERK**  mitogen activate protein kinase extracellular signal related kinase activation inhibit  **NMDA**  receptor dependent long term potentiation LTP.  Ref: 12736339 J Neurosci, 2003
- Consistent with the notion that  **ERK**  activation by  **NMDA**  receptor stimulation is PI3 kinase dependent, the PI3 kinase inhibitor wortmannin partially inhibited ERK2 activation induced by bath application of  **NMDA**  and strongly suppressed ERK2 activation by high frequency synaptic stimulation.  Ref: 12736339 J Neurosci, 2003
- In addition,  **NMDA**  receptor activation of  **ERK**  and PKB may occur through overlapping signaling pathways that bifurcate at the level of Ras.  Ref: 12358757 J Neurochem, 2002
- **NMDA**  10 0 6 M induced vasodilation was reversed to vasoconstriction following fluid percussion brain injury, but such responses were partially restored by genistein, 7 trihydroxy isoflavone, U0126 diamino dicyano bis 0 aminophenylmercapto butadiene and SB203580 4 4 fluorophenyl 2 4 methylsulfinylphenyl 5 4 pyridyl 1H imidazole, PTK,  **ERK**  and p38 MAPK inhibitors 9 1% and 16 1%, sham control.  Ref: 12921870 Eur J Pharmacol, 2003
- Our results suggest that both  **NMDA**  and non  **NMDA**  receptors are involved in supraspinally administered KA induced pyramidal cell death in CA3 region of hippocampus in the mouse and the p  **ERK**  and the dephosphorylation of CREB protein may play an important role in CA3 region cell death of the hippocampus induced by KA administered supraspinally.  Ref: 12521295 Mol Cells, 2002
- This study investigated the contribution of activation of protein tyrosine kinase PTK and the extracellular signal regulated kinase  **ERK**  and p38 isoforms of mitogen activated protein kinase MAPK in impaired vasodilation to  **NMDA**  after fluid percussion brain injury in pigs equipped with a closed cranial window.  Ref: 12921870 Eur J Pharmacol, 2003
- PI3 kinase inhibitors also block activation of  **ERK**  by  **NMDA**  receptor stimulation, suggesting that PI3 kinase inhibitors block LTP because PI3 kinase is an essential upstream regulator of  **ERK**  activation.  Ref: 12736339 J Neurosci, 2003
- Glutamate transmission via  **NMDA**  and mGluR1 but not AMPA receptors was critical to this  **ERK**  activation.  Ref: 14572451 Mol Cell Neurosci, 2003
- Activation of  **ERK**  and Akt signaling in focal cerebral ischemia modulation by TGF alpha and involvement of  **NMDA**  receptor.  Ref: 12586553 Neurobiol Dis, 2002
